# A Bit of Fit: Minimalist Intervention in Adolescents Based on a Physical Activity Tracker

**DOI:** 10.2196/mhealth.7647

**Published:** 2017-07-06

**Authors:** Jeffrey Gaudet, François Gallant, Mathieu Bélanger

**Affiliations:** ^1^ Centre de formation médicale du Nouveau-Brunswick Moncton, NB Canada; ^2^ Faculty of Medicine and Health Sciences Université de Sherbrooke Sherbrooke, NB Canada; ^3^ Vitalité Health Network Moncton, NB Canada

**Keywords:** health behavior, health promotion, mHealth, physical activity tracker

## Abstract

**Background:**

Only 5% of Canadian youth meet the recommended 60 minutes of moderate to vigorous physical activity (MVPA) per day, with leisure time being increasingly allocated to technology usage. Direct-to-consumer mHealth devices that promote physical activity, such as wrist-worn physical activity trackers, have features with potential appeal to youth.

**Objective:**

The primary purpose of this study was to determine whether a minimalist physical activity tracker-based intervention would lead to an increase in physical activity in young adolescents. A secondary aim of this study was to assess change in physical activity across a 7-week intervention, as measured by the tracker.

**Methods:**

Using a quasi-experimental crossover design, two groups of 23 young adolescents (aged 13-14 years) were randomly assigned to immediate intervention or delayed intervention. The intervention consisted of wearing a Fitbit-Charge-HR physical activity tracker over a 7-week period. Actical accelerometers were used to measure participants’ levels of MVPA before and at the end of intervention periods for each group. Covariates such as age, sex, stage of change for physical activity behavior, and goal commitment were also measured.

**Results:**

There was an increase in physical activity over the course of the study period, though it was not related to overall physical activity tracker use. An intervention response did, however, occur in a subset of participants. Specifically, exposure to the physical activity tracker was associated with an average daily increase in MVPA by more than 15 minutes (*P*=.01) among participants who reported being in the action and maintenance stages of behavior change in relation to participation in physical activity. Participants in the precontemplation, contemplation, and preparation stages of behavior change had no change in their level of MVPA (*P*=.81).

**Conclusions:**

These results suggest that physical activity trackers may elicit improved physical activity related behavior in young adolescents demonstrating a readiness to be active. Future studies should seek to investigate if integrating physical activity trackers as part of more intensive interventions leads to greater increases in physical activity across different levels of stages of behavior change and if these changes can be sustained over longer periods of time.

## Introduction

Despite the documented benefits of physical activity on the physical, psychological, and social well-being of young people [1,2], only 5% of Canadian youth between the ages of 12 and 17 meet the recommended guidelines of 60 minutes of moderate to vigorous physical activity (MVPA) per day [3]. This is worrisome as physical inactivity during youth has been shown to track into adulthood [4] and lead to an increased risk for multiple chronic conditions [5]. The need to identify successful interventions aimed at increasing physical activity among youth is warranted, given that results from previous intervention studies have shown room for improvement [6].

As youth are allocating increasingly more time to technology [7], technological platforms have concurrently gained popularity as a means to target health behavior [8]. mHealth technologies, including wearable physical activity trackers and mobile apps could be promising components of interventions aimed at reducing physical inactivity [9,10]. Past research has shown that interventions centered on the use of simple wearable devices, such as uniaxial pedometers, can lead to increases in physical activity participation and reductions in body mass index and blood pressure [11,12]. However, the strongest intervention effects have generally occurred when technological platforms were combined with at least one theoretically-based behavior change component [11]. Given that the newest accelerometer-based physical activity tracking devices are equipped with user-friendly features that relate to behavior change theory such as goal setting, review of past goals, and frequent feedback, it is possible that their effects on adhering to healthy physical activity levels is stronger [13]. The effects of such devices may also be more important in some sub-groups, for example, based on the stages of readiness to change. Past research has suggested that there is greater potential for intervention effectiveness in increasing physical activity among people who demonstrate the highest levels of readiness to get active as compared with those closed to the idea of being more physically active [14,15].

The use of commercially available accelerometer-based physical activity trackers as a means to target behavior change has been linked to increases in daily steps and time spent in MVPA in various adult populations [16-18]. Studies involving youth have, however, been limited to patients living with disease [19-21], eight-year-old children [22], and urban youth living in an under-resourced community [23]. Thus, the primary purpose of this study was to determine if a minimalist physical activity tracker-based intervention would lead to an increase in physical activity in young adolescents. Secondary aims of this study were to assess change in physical activity across a 7-week intervention, as measured by the physical activity tracker, and to assess differences in change in physical activity based on individuals’ goal commitment and stage of behavior change.

## Methods

### Participants and Procedures

**Figure 1:**
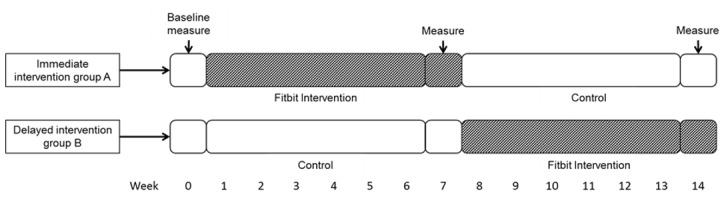
Crossover design of the physical activity tracker-based intervention study.

All students in the two Grade 8 (13-14 years old) classes in one school were invited to participate in this study. To participate, students had to obtain written informed consent from parents or legal guardians and provide assent as approved by the Centre Hospitalier de l’Université de Sherbrooke ethics committee. This study employed a quasi-experimental crossover design with a 7-week physical activity tracker-based physical activity intervention and a control period. Participants in one class (group A) were randomly assigned to the immediate intervention group and participants in the other class (group B) were assigned to the delayed intervention group (Figure 1). The crossover design was used to allow participants from both groups to have a chance to experiment with the physical activity tracker. This design also allowed controlling for individual-level covariates as participants served as their own control. Baseline measurements were obtained before the first week of intervention. Physical activity measurements for all participants were also obtained at the end of both intervention periods: weeks 7 and 14. This study took place between February and June, 2016.

### Intervention

The intervention focused on increasing physical activity via self-monitoring and self-regulation. During the intervention period, each participant was provided a wrist-worn physical activity tracker (Fitbit, model Charge HR; FitBit Inc. San Francisco, USA) that was equipped with a small screen displaying real-time summary data for steps, heart rate, distance, calories, and stairs climbed. Physical activity intensity minutes and temporal patterns were also available to participants through the accompanying Web-based Fitbit user account. Individual Fitbit user accounts were prepared by the study coordinator for each participant before the distribution of the devices. The study coordinator was also responsible for demonstrating how to use the Fitbit device and Web-based Fitbit user account. The intervention was similar to that used in previous studies [10,16] and was based on Behavior Change Technique research [24]. Empirical research shows that self-monitoring, in combination with at least one other self-regulation skill, can lead to positive physical activity related behavior change [24]. An individualized goal that can be tracked using measures provided by the physical activity tracker was set by each participant, following a brief researcher-led presentation of the SMART (specific, measurable, attainable, realistic, and timely) principles of goal-setting [25]. Beyond receiving physical activity trackers during the intervention period and being asked to select a goal, participants did not receive other interventions. During the control period, participants received no intervention.

### Measures

Actical accelerometers (Philips - Respironics, Oregon, USA) were worn on the hip for seven days at baseline, week 7, and week 14. This lightweight omnidirectional accelerometer has been validated as an objective measure of physical activity in youth aged 10 to 15 years [26]. Accelerometer data were recorded in 15-second intervals, then cleaned and managed using procedures recommended by Statistics Canada [27] through a series of publicly available SAS codes adapted for this type of study [28]. Time spent in different physical activity intensities were determined by using cut-points established in previous research involving Actical accelerometers in children [29]. Sedentary activity corresponded to count values below 100, light physical activity to counts between 100 and 1500, moderate physical activity to counts between 1500 and 6500, and vigorous physical activity to counts greater than 6500. Only data for valid days, defined as 10 hours or more of wear time, were retained for analyses. Daily averages for MVPA were calculated from valid days.

Using the Fitabase analytics system (Small Steps Labs, San Diego, CA, USA), data from all physical activity trackers were remotely collected and aggregated whenever data were transmitted to users’ personal Fitbit dashboards. Data captured included heart rate, daily steps, and minutes of intensity-specific physical activity. Wear time was calculated by subtracting non-wear time from 24 hours and non-wear time was defined as any interval with at least 60 consecutive seconds of zero recording of heart rate. As heart rate was recorded at variable time periods by the physical activity tracker, allowing for 60 consecutive seconds of zero recording of heart rate was sufficient to distinguish non-wear from wear time. Indeed, identification of valid days remained stable across use of higher thresholds, for example 5, 15, 30, and 60 minutes, whereas significant information was lost with a threshold set under 60 seconds.

A baseline questionnaire was used to collect information regarding age, sex, stage of change for physical activity behavior, and goal commitment. Specifically, participants indicated whether they participated in at least 60 minutes of physical activity per day, using an item corresponding to the five stages of behavior change (precontemplation, “No, I do not participate in physical activity and I do not intend to in the next 6 months;” contemplation, “No, I do not participate in physical activity regularly but I intend to in the next 6 months;” preparation, “No, I do not participate in physical activity regularly but I intend to in the next 30 days;” action, “Yes, I have been participating in physical activity regularly, but for less than 6 months;” maintenance, “Yes, I have been participating in physical activity regularly for more than 6 months”) [30,31]. For analyses, these five stages of behavior change were grouped into two categories representing adoption (ie, action and maintenance) and preadoption (ie, precontemplation, contemplation, and preparation) as done by De Bourdeaudhuij et al [32]. Goal commitment, defined as determination to attain an objective, was assessed using a five-item scale refined and validated by Klein et al [33]. In this scale, participants indicated their level of commitment to the personal goal they had set with the following items using a 5-point Likert scale: (1) It’s hard to take this goal seriously; (2) Quite frankly, I don’t care if I achieve this goal or not; (3) I am strongly committed to pursuing this goal; (4) It wouldn’t take much to make me abandon this goal; (5) I think this is a good goal to shoot for. Items 1, 2, and 4 were reverse-scored before calculating a mean of the five items meant to represent the construct of goal commitment [33].

### Statistical Power Calculation

Based on previous research, which showed that a similar Fitbit-based physical activity tracker intervention induced a 36% increase in MVPA (pre-post change mean=172, SD=83 to mean=234, SD=119), albeit in a sample of adult women [16], we estimated that 22 participants per group would provide 80% power with 95% CIs.

### Data Analysis

Wilcoxon rank-sum tests were used to compare between group differences at baseline and following both intervention periods. Wilcoxon signed-rank tests were used to compare within group difference at different time points. A multiple linear regression model was used to assess pre-post change in physical activity while controlling for the effects of time, goal commitment, and stage of behavior change. Physical activity data, from both physical activity trackers and accelerometers, were adjusted for valid wear days. A valid wear day was defined as at least 10 hours of wear time. Repeated measures analysis and Tukey post-hoc tests were used to assess changes in physical activity tracker-measured physical activity throughout the intervention period. All analyses were conducted using SAS 9.4 (SAS Institute, Inc., Cary, North Carolina, USA).

## Results

We recruited 46 of the 52 students eligible for this study. On average, the participants (52% girls, 24/46) were 13 years (SD=0.34) old and age and gender were distributed equally in both groups. There were no apparent or statistical (at Cronbach alpha<.05 with Fisher’s exact test or independent *t* tests) differences in baseline characteristics between the two study groups (Table 1). At baseline, participants wore the accelerometer for an average of 13.0 (SD=1.3) hours per day and performed a mean of 35.5 (SD=19.0) minutes of MVPA per day. Accelerometer data were available for analyses for 43 participants at baseline, 32 at the end of the first intervention period (week 7), and 27 at the end of the second intervention period (week 14).

**Table 1:** Baseline characteristics of study participants.

Characetristics		Group A (n=23)	Group B (n=23)
Age in years, mean (SD)		13 (0.3)	13 (0.4)
Females, n (%)		12 (52)	12 (52)
Accelerometer Valid Days, mean (SD)		4.7 (1.7)	3.1 (2.1)
Accelerometer Wear Time in hours, mean (SD)		13.4 (1.2)	12.6 (1.3)
MVPA^a^ in minutes, mean (SD)		34.7 (19.1)	36.2 (19.2)
Sedentary Time in minutes, mean (SD)		623.6 (82.6)	587.5 (67.1)
Goal commitment score from 1 to 5, mean (SD)		4.1 (0.6)	3.7 (0.9)
**Stage of behavior change, n (%)**				
	Precontemplation	3 (13)	1 (4)
	Contemplation	1 (4)	4 (17)
	Preparation	2 (9)	4 (17)
	Action	3 (13)	5 (22)
	Maintenance	14 (61)	9 (38)

^a^MVPA: Moderate to vigorous physical activity.

**Figure 2:**
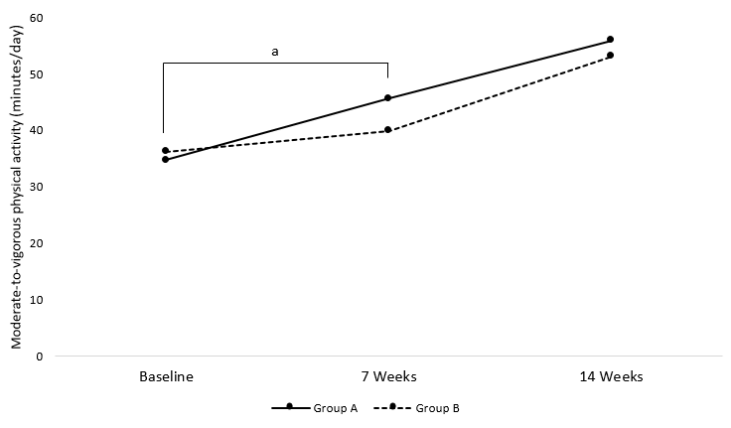
Baseline to 14-week changes in objectively measured physical activity from Actical accelerometers (group A was exposed to Fitbit from week 1 to 7 and group B was exposed to Fitbit from week 8 to 14, and “a” indicates significant difference between pre and post measurements within group A: *P*=.03).

In the main analysis, the multiple regression model showed no overall effect of wearing the physical activity tracker on MVPA levels; however, a positive effect of time was found (*P*=.008). However, relative to baseline, the first group to receive the physical activity tracker intervention, group A, increased MVPA by 10.9 minutes/day (*P*=.03) over the first 7-week period, whereas the increase in MVPA for the delayed intervention group, group B, corresponded to 3.7 minutes/day (*P*=.56) during the same period (Figure 2). During weeks 8 to 14, group B was exposed to the intervention and displayed an average increase of MVPA of 13.2 minutes/day (*P*=.49), while the increase in MVPA represented 10.3 minutes/day (*P*=.64) in group A for this second period. There was no significance between group differences at the baseline or at the 7 weeks or 14 weeks assessments.

**Figure 3:**
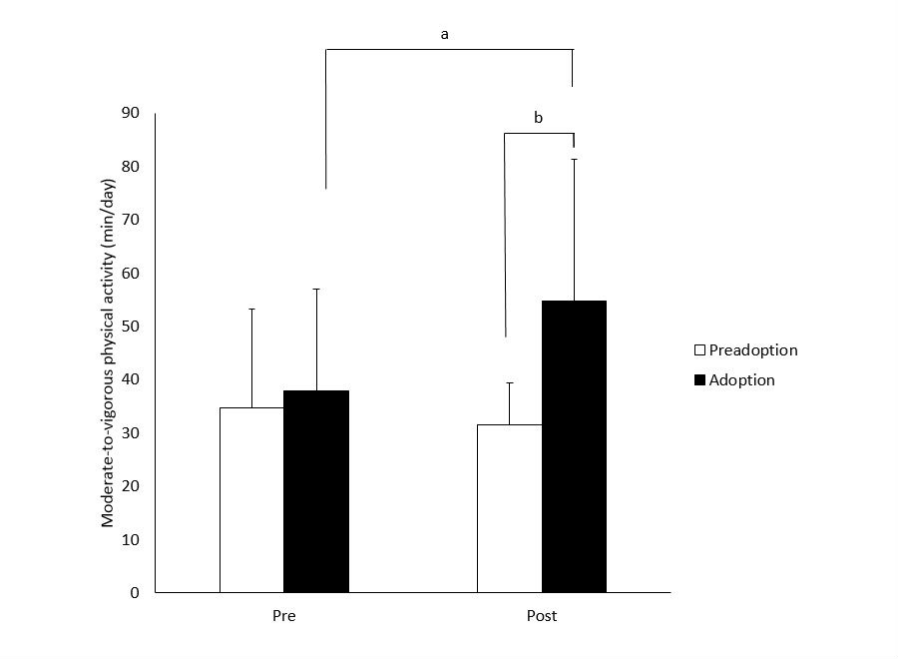
Moderate-to-vigorous physical activity among preadoption (precontemplation, contemplation, and preparation) and adoption (action and maintenance) participants before and after a 7-week minimalist physical activity tracker intervention: data are means and standard deviations (SD), “a” indicates significant differences between pre and post measurements within group (P=.01) based on the Wilcoxon signed-rank test, and “b” indicates significant difference between groups at the post-intervention measurement (P=.02) based on the Wilcoxon sum-rank test.

After combining the pre and post intervention scores of both groups (group A pre-intervention at week 0 and post-intervention at 7 weeks, and group B pre-intervention at 7 weeks and post-intervention at 14 weeks), Wilcoxon tests suggested that changes in mean MVPA were related to differences in stages of behavior change (Figure 3). Participants in the adoption stages had a significant increase in MVPA from pre to post-intervention (*P*=.01), whereas participants in the preadoption stages did not change (*P*=.81). Whereas both groups had similar levels of MVPA at the pre-intervention time point, the post-intervention difference between the adoption and preadoption group was over 23 minutes of MVPA (*P*=.02). Moreover, physical activity tracker data showed that participants in the adoption stages averaged 2900 more steps and 20 more minutes of daily physical activity during the intervention phase than those in the preadoption stages. No association was found between goal commitment and MVPA.

**Figure 4:**
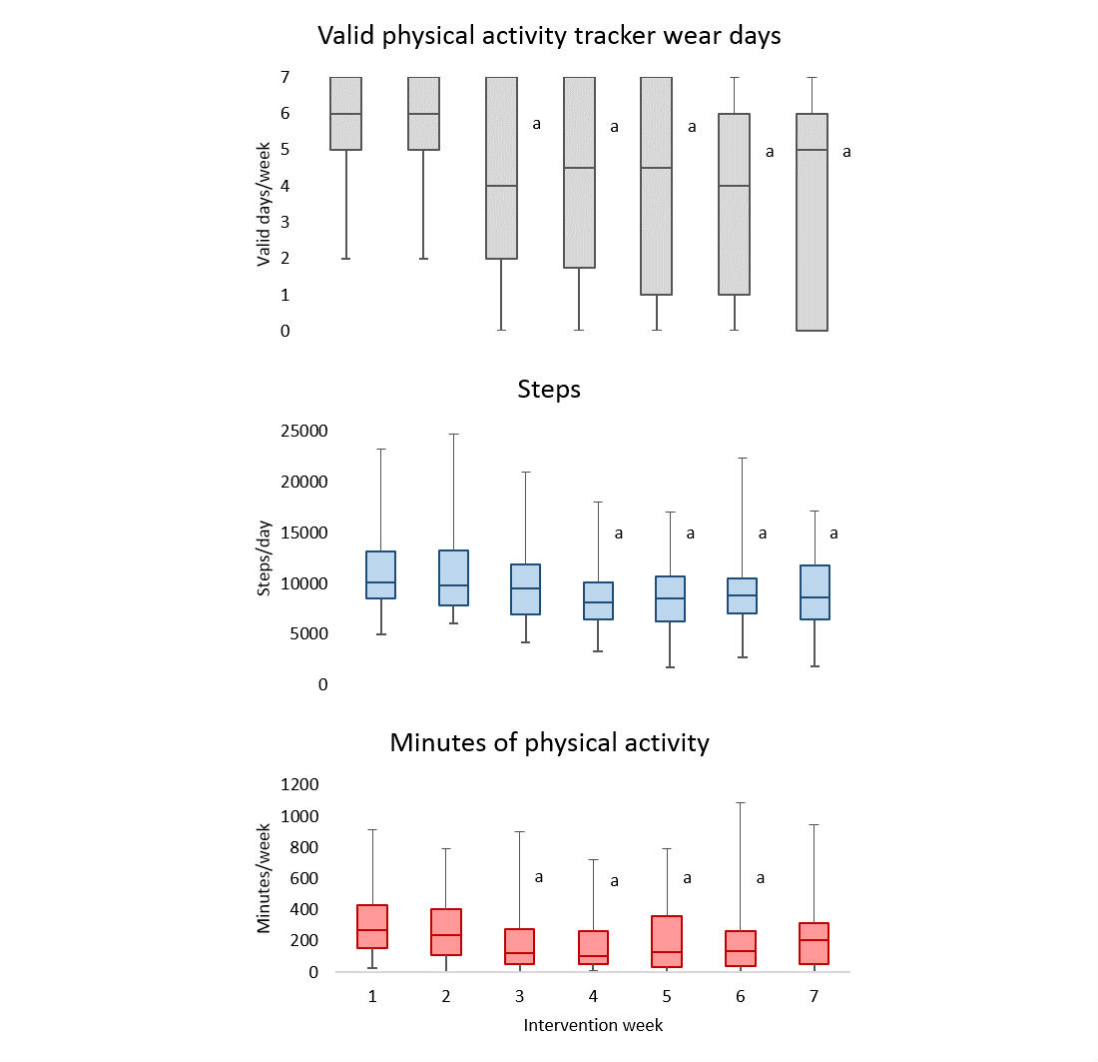
Changes in physical activity tracker measures during the 7-week intervention period (Valid days are defined as those with 10 hours or more of wear time, minutes of physical activity are Fitbit-defined minutes of “fairly to very active” physical activity, median and quartiles are represented in box plots, and “a” indicates significant differences from initial week).

The median participant in this study wore the physical activity tracker device for at least 10 hours per day on 67.3% of intervention days (33/49). Mean valid wear period was 30 days (SD=13), with a range of 6 to 49 days. Tukey post-hoc investigations suggest that wearing of the physical activity tracker peaked during the first two weeks of the intervention period and then dropped abruptly at the third week (Figure 4). The mean number of valid wear days during weeks 3 to 7 was significantly lower than in the first two weeks (*P*<.001). Physical activity tracker measured physical activity time and step count also showed similar decline after week 2 (*P*=.04) and week 3 (*P*=.01), respectively.

## Discussion

### Principal Findings

With little research to date on the effects of using direct-to-consumer mHealth trackers as behavior change tools, the current study sought to examine the effectiveness of using a minimalist physical activity tracker-based intervention as a means of encouraging increased physical activity in adolescents. Although the main results suggested no change in MVPA as a result of having been exposed to a physical activity tracker, secondary analyses suggest that the interventions may have had beneficial effects for some sub-groups. For example, an intervention response occurred in a subset of participants who reported being in the action and maintenance stages of behavior change (adopters) in relation to participation in physical activity. Whereas these participants increased their daily average of MVPA by more than 15 minutes during the intervention period, those in the precontemplation, contemplation, and preparation stages (preadopters) had no change in their level of MVPA. This distinction manifested itself without the intervention being intentionally tailored to any specific stage of behavior change. Nevertheless, previous studies show that psychosocial determinants positively associated with physical activity generally increase across the stages of behavior change [32,34]. Adolescents in the adoption stages typically perceive fewer barriers, more benefits and have a better attitude and more self-efficacy in relation to physical activity participation [32]. Such underlying conditions likely predisposed these participants to be more receptive and to respond favorably to the exposure to a physical activity tracker. Although minimalist in nature, the introduction of a physical activity tracker may therefore represent a sufficient trigger for youth in the adoption stages to increase their level of physical activity. However, the intervention was likely too simple to induce a behavior change among youth in the preadoption stages. Stage-specific intervention research suggests that in order to successfully motivate individuals in the preadoption stages, it is necessary to consider cognitive aspects of behavior change such as raising consciousness, social liberation, self-re-evaluation, self-liberation and counter-conditioning, helping relationships, and reward management [35,36].

The increase in physical activity among adopters may also be attributable to the fact that there was room for growth. Despite perceiving themselves as being active, objective measures suggested that participants in the adoption stages were not more active than those in the preadoption stages at baseline. This is similar to results from another study, which found little to no difference among levels of objectively measured MVPA of adolescents at different stages of behavior change [30]. Our results therefore point to the potential usefulness of assessing readiness to change before intervening. Although preadoption and adoption adolescents presenting similar objectively measured physical activity at baseline, the simple one-item questionnaire used to assess stages of change behavior change in this study correctly pre-identified participants who would best respond to the introduction of a minimalist physical activity tracker-based intervention.

This study was initiated during the middle of winter and extended to the end of spring. Thus, the start and end points of the study coincide with the typical periods of lowest and highest annual levels of physical activity in this age group, respectively [37-39]. This is noteworthy as the physical activity tracker-based intervention elicited an increase in physical activity during the study phase that corresponded to winter. Although it needs to be corroborated by other studies, it is possible that the introduction of physical activity trackers during this season could help some adolescents increase physical activity during the colder winter months. This is in support of findings from Dean et al [19] who observed, among a sample of adolescents with congenital heart disease, that wearing a physical activity tracker, as compared with not wearing one, was associated with a less abrupt decline of physical activity during winter months.

Continuous objective measurements obtained from physical activity trackers provided information suggesting that there was an acute effect of receiving the physical activity tracker. Specifically, compliance to wearing the physical activity tracker was at its highest during the first weeks of intervention. This would suggest that the device had a novelty effect, as demonstrated by others [23,40,41]. For instance, Shih et al [40] measured 50% attrition rates after the 2 week mark in a 6-week study in undergraduate students, while Schaefer et al [23], had only 2 participants (8%) use their physical activity trackers for a 4 month follow-up study. Beyond a decrease in compliance, it was noted that the average number of daily steps and minutes of physical activity were also at their peaks early in the intervention period. During the first three weeks, participants averaged between 9800 and 12,000 steps per day, which is close to the 10,000 to 12,000 steps per day recommended for this age group [42-44]. After the third week, however, this number declined to less than 9000 steps per day. Whereas normative data indicate that the majority of adolescents do not meet the step count recommendation [44], our findings suggest that there may be potential for physical activity trackers to encourage adolescents to perform near recommended levels of physical activity, at least over a short period of time.

It is possible that accompanying the distribution of physical activity trackers with a more intensive intervention would have led to greater compliance in wearing the device and greater increases in physical activity. The participants in our study, nevertheless, wore physical activity trackers to a greater extent than adolescents from under-resourced communities in another study [23], but also considerably less than post-menopausal women in another study [16]. Direct comparison to wear time during intervention in other studies involving adolescents is not possible as this kind of information tends not to be reported [41,45].

### Limitations

Limitations of this study need to be considered when interpreting the results. First, even though none of the participants was lost during the study, there was an unanticipated decline in compliance in wearing accelerometers at both post-intervention periods. This contributed to a loss of power to detect meaningful differences in physical activity, despite having initially recruited enough participants for adequate power. This drop in compliance may also have contributed to selection bias, wherein participants least likely to become more active did not wear the device at follow-up periods. Research is warranted to better understand adolescent engagement toward physical activity trackers to develop tailored interventions aimed at increasing compliance in this population [41,46]. Comparison between participants who completed all three evaluation periods and those who did not revealed no significant differences in physical activity level at baseline or distribution in stages of behavior change. Second, it needs to be considered that some activities were not measured because the accelerometer or physical activity tracker could not be worn (eg, swimming). Third, caution must be taken in interpreting physical activity tracker measured physical activity data as the proprietary algorithms used to calculate minutes of physical activity at different intensities are not publicly available. Fourth, although our results revealed a difference in intervention response between adolescents in the preadoption and adoptions stages, the small sample did not allow for in-depth analyses between each stage. Given the theoretical and empirical evidence of psychosocial and processes of change differences between each of the stages, future research with a larger sample is warranted to help elucidate which stages benefit the most from mHealth devices such as physical activity trackers. Future research should also assess whether similar physical activity tracker-based interventions lead to progressions in the stages of behavior change even among individuals who do not change their level of MVPA. Finally, although this study benefited from the strengths of randomization and crossover, having more randomization units in future studies would help attain group similarities even among unmeasured potentially confounding variables.

### Conclusions

In summary, although no overall effect was found, the secondary results of this study suggest that there is potential value in using physical activity trackers to increase physical activity among adolescents in adoption stages of behavior change related to participation in physical activity. Adolescents in the adoption stages of behavior change may benefit from simply gaining access to a direct-to-consumer mHealth device designed to track physical activity. Future research is needed to better understand what additional strategies could be paired with physical activity trackers to lead to improvements in physical activity levels of adolescents in all stages of behavior change. This study also has implications for research as it demonstrates the feasibility of continuously and objectively measuring physical activity during an intervention involving adolescents.

## Abbreviations

MVPA: moderate to vigorous physical activity
SMART: specific, measurable, attainable, realistic, and timely
